# Magnetic Resonance Spectroscopy Detectable Metabolomic Fingerprint of Response to Antineoplastic Treatment

**DOI:** 10.1371/journal.pone.0026155

**Published:** 2011-10-12

**Authors:** Alessia Lodi, Sabrina M. Ronen

**Affiliations:** Department of Radiology and Biomedical Imaging, University of California San Francisco, San Francisco, California, United States of America; Instituto de Investigación Sanitaria INCLIVA, Spain

## Abstract

Targeted therapeutic approaches are increasingly being implemented in the clinic, but early detection of response frequently presents a challenge as many new therapies lead to inhibition of tumor growth rather than tumor shrinkage. Development of novel non-invasive methods to monitor response to treatment is therefore needed. Magnetic resonance spectroscopy (MRS) and magnetic resonance spectroscopic imaging are non-invasive imaging methods that can be employed to monitor metabolism, and previous studies indicate that these methods can be useful for monitoring the metabolic consequences of treatment that are associated with early drug target modulation. However, single-metabolite biomarkers are often not specific to a particular therapy. Here we used an unbiased 1H MRS-based metabolomics approach to investigate the overall metabolic consequences of treatment with the phosphoinositide 3-kinase inhibitor LY294002 and the heat shock protein 90 inhibitor 17AAG in prostate and breast cancer cell lines. LY294002 treatment resulted in decreased intracellular lactate, alanine fumarate, phosphocholine and glutathione. Following 17AAG treatment, decreased intracellular lactate, alanine, fumarate and glutamine were also observed but phosphocholine accumulated in every case. Furthermore, citrate, which is typically observed in normal prostate tissue but not in tumors, increased following 17AAG treatment in prostate cells. This approach is likely to provide further information about the complex interactions between signaling and metabolic pathways. It also highlights the potential of MRS-based metabolomics to identify metabolic signatures that can specifically inform on molecular drug action.

## Introduction

The Warburg effect, wherein cancer cells have an abnormally elevated rate of glucose consumption and aerobic glycolysis, was first discovered in the 1920s [1]. Research over the past decade is increasingly demonstrating that several other aspects of metabolism are also profoundly different in cancer cells [2], [3]. Many of these changes appear to result from the acquisition of mutations that develop during oncogenesis and provide a growth advantage to the cancerous cells in the tumor microenvironment. Knowledge of these metabolic changes is now being used as the basis for development of more specific molecular imaging methods. For instance, the higher glycolytic demand characterizing cancer cells has been exploited in [^18^F] 2-fluoro-2-deoxy-D-glucose positron emission tomography (FDG-PET) imaging of tumors [4]. In recent years, owing to advances in dynamic nuclear polarization (DNP), the elevated glycolytic rates in tumors have also been imaged using ^13^C magnetic resonance spectroscopy (MRS) by probing the conversion of hyperpolarized pyruvate into lactate [5]–[7]. Conversely, several reports have been published reporting normalization of glucose metabolism as an indication of response to targeted treatment [8], [9]. A decrease in pyruvate to lactate conversion in response to treatment with phosphoinositide 3-kinase (PI3K) or receptor tyrosine kinase (RTK) inhibitors was shown in different tumor types by ^13^C MRS [10], [11]. Phosphocholine (PC) or, clinically, total choline (tCho, comprised of choline, PC and glycerophosphocholine) was also identified in several MR studies as an important biomarker that is generally elevated in cancer cells and associated with more aggressive and invasive phenotypes [12]–[20]. Inhibition of cell proliferation following treatment with targeted therapies, including inhibitors of Ras, PI3K, mitogen-activated protein kinases (MAPK) and hypoxia inducible factor (HIF) led in most cases to a drop in PC and tCho [21]–[31]. In particular, inhibition of HIF-1α with PX-478 in HT-29 colorectal cancer xenografts induced a drop in intracellular PC levels as well as tCho [26]. Pharmacological intervention with the PI3K inhibitor PI-103 induced a drop in PC in PC3 prostate cancer cells and HCT116 colorectal cancer cells [21]. Similarly in MDA-MB-231, MCF-7 and Hs578T breast cancer cells treated with either LY294002 or Wortmannin PC dropped [22]. Finally orthotopic glioblastoma tumors treated with the PI3K inhibitor PX-886 also lead to a drop in tCho [31]. Interestingly, treatment with the heat shock protein (HSP) 90 inhibitor 17-(Allylamino)-17-demethoxygeldanamycin (17AAG), which likely has a more complex effect on cellular signaling as it targets a number of protein kinases (including Akt, MEK and c-Raf) as well as hormone receptors [32], was reported to cause an increase in PC in several cancer models including breast and colorectal [24], [25]. However, treatment with 17AAG in prostate cancer xenografts in mice (hormone sensitive CWR22 and hormone resistant CWR22r) induced a drop in the tCho pool [27]. While demonstrating the value of metabolic changes as biomarkers of response to targeted therapies, these studies also highlight the fact that some metabolic changes are common to different therapeutic agents. An alternative approach is the use of completely untargeted and global metabolic profiling methods coupled with a robust chemometric analysis based on multivariate statistical methods of analysis. These approaches have been proposed for the detection and identification of global metabolic changes in human biofluids as a diagnostic tool [33]–[35]. Similar approaches have also been employed in cell model systems to investigate the metabolic effects of drug treatments or different genetic phenotypes [36]–[38]. Recently this approach was also used in studies of tumor biopsy samples providing a method to distinguish between normal and malignant tissue in different cancer types [39]–[43]. Due to the completely untargeted nature of these studies and the size and complexity of the metabolic signature datasets, the application of appropriate multivariate statistical methods of analysis is key to identifying the most prominent changes in the metabolic signature. In cell model systems principal component analysis (PCA) is usually appropriate to efficiently identify and discriminate the underlying metabolic variation in the datasets. Moreover, PCA is a completely unsupervised method and does not require any *a priori* information about the data allowing for a completely unbiased analysis of the datasets [44].

Several PI3K inhibitors are currently in clinical trials for cancer treatment [45]. Similarly, the HSP90 inhibitor 17AAG has been tested in the clinic for treatment of solid tumors [46], [47]. We were therefore interested in assessing the individual signatures of response that could potentially be translated into the clinic. In this study, we investigated two prostate cancer cell lines, PC3 and LNCaP, and used an untargeted and unbiased ^1^H MRS-based metabolomics approach to investigate the metabolic consequences of pharmacological inhibition of the PI3K signaling pathway and the HSP90 protein chaperone using LY294002 and 17AAG, respectively. Moreover, to confirm the generality of our findings, we investigated the metabolic changes induced by LY294002 and 17AAG in MCF-7 breast cancer cells. Based on the analysis of the comprehensive changes in the metabolome of these cell lines we identified a pattern of metabolic changes that was different for each of the two drugs but identical for the three different cell lines. This approach could provide drug-specific metabolic readouts of molecular drug action. Furthermore, this method could be used to identify previously unrecognized metabolic changes associated with modulation of specific signaling pathways.

## Results

### Treatment doses and target inhibition in prostate cancer cell lines

**Figure 1:**
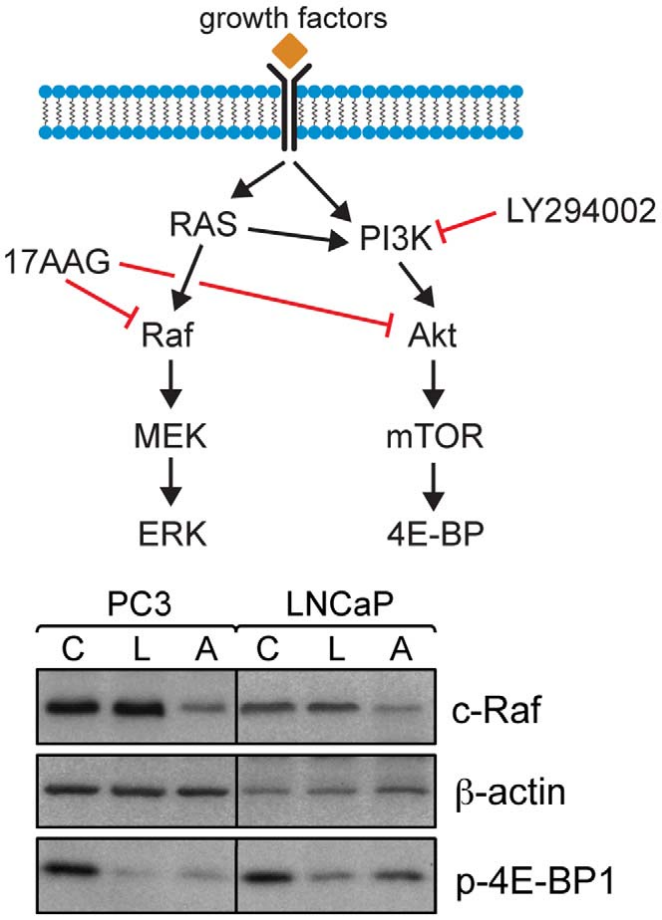
Inhibition of target signaling pathways in cancer cells following drug treatment. Schematic of signaling pathways targeted by LY294002 and 17AAG and Western blots showing modulation of p-4E-BP1 and c-Raf (β-actin as loading control) levels following administration of DMSO (solvent control; C), LY294002 (L) or 17AAG (A).

Two prostate cancer cell lines, PC3 and LNCaP, were treated for 48 hours with LY294002, a PI3K inhibitor, and 17AAG, a HSP90 inhibitor. For each cell line, the treatment doses were determined such that the cell viability remained approximately constant during treatment (simulating tumor stasis during treatment, as opposed to the control cells which proliferate normally). These doses were 25 µM and 10 µM LY294002, and 1 µM and 0.25 µM 17AAG for PC3 and LNCaP prostate cancer cells, respectively. Inhibition of the target proteins at the determined doses was confirmed by Western blotting. Following 48 hours of treatment with LY294002, inhibition of the PI3K signaling pathway was confirmed in both PC3 and LNCaP cells by probing for p-4E-BP1 protein levels, which decreased following treatment (**Fig. 1**). The effectiveness of the 48 hours 17AAG treatment was verified by probing the levels of the HSP90-client protein c-Raf, which decreased in both cell lines (**Fig. 1**). Moreover, following 17AAG treatment, p-4E-BP1 decreased to intermediate levels between the control and LY294002 treated samples (**Fig. 1**).

### Proton MRS-based metabolomics analysis in prostate cancer cells

**Figure 2:**
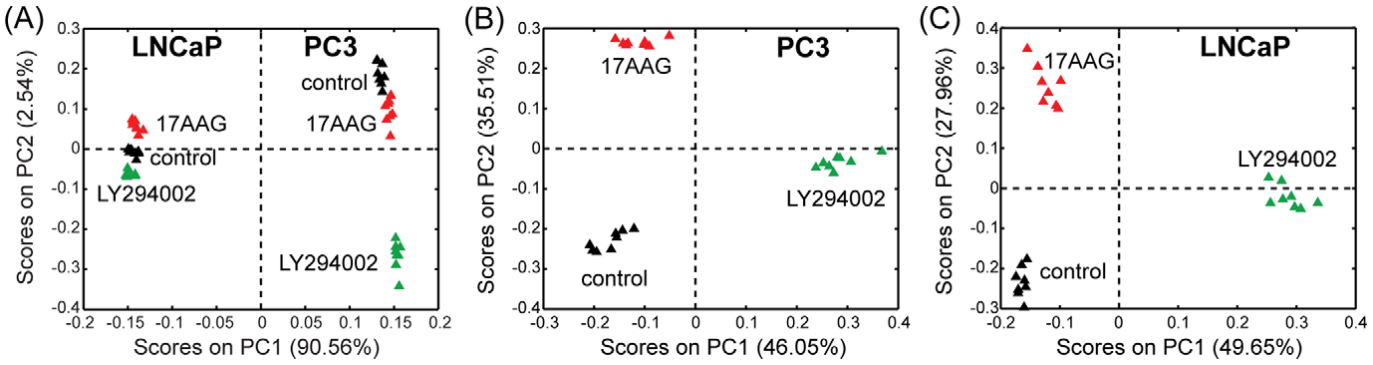
Multivariate statistical analysis of the MR spectra. Scores plots (PC1 vs PC2) obtained by performing PCA on the MR spectra acquired on polar extracts (8 replicates per treatment condition) of (A) both PC3 and LNCaP cells, and individual (B) PC3 and (C) LNCaP prostate cancer cells following a 48-hrs treatment with DMSO (solvent control, black), LY294002 (green) and 17AAG (red).

**Figure 3:**
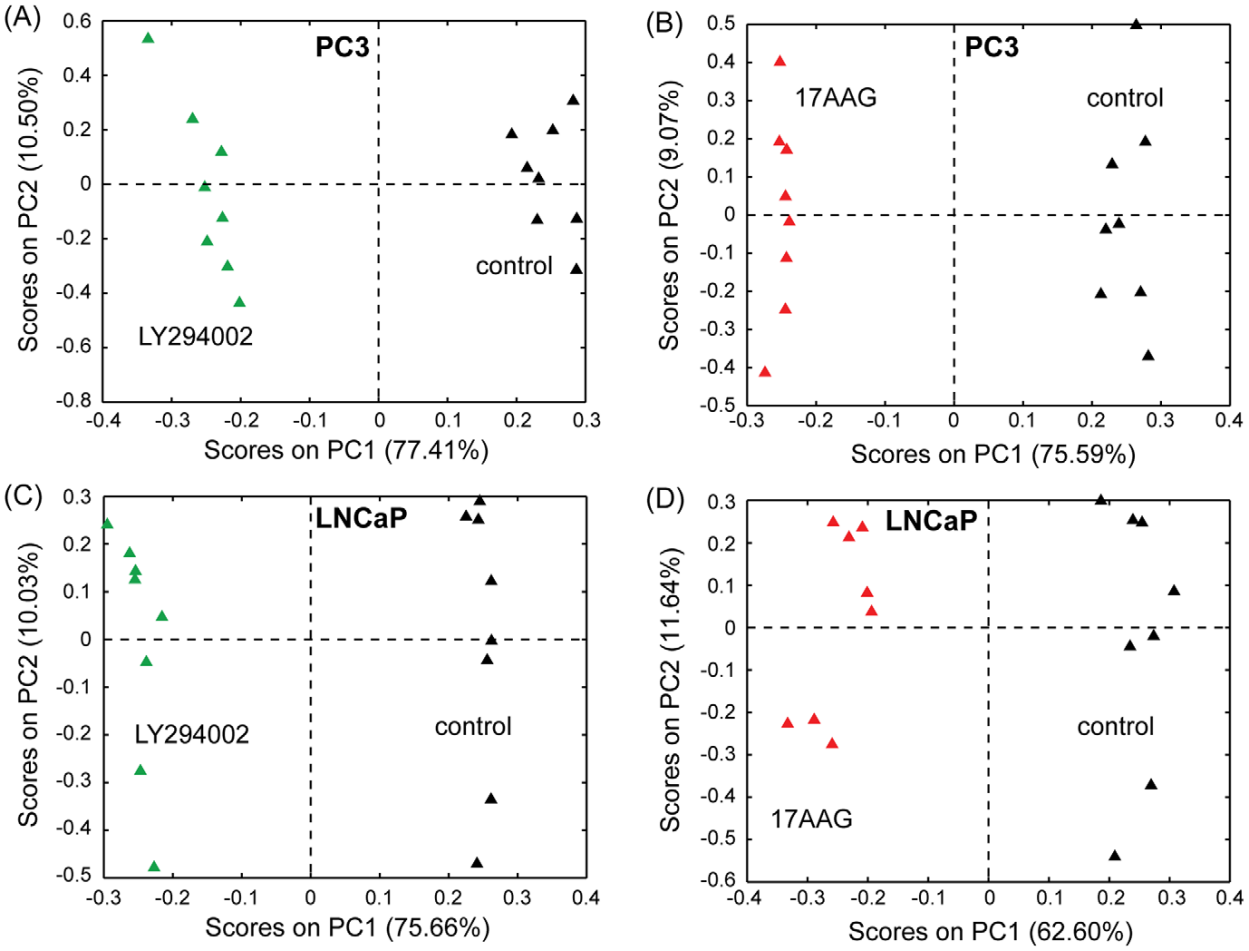
Multivariate statistical analysis of the MR spectra. Scores plots (PC1 vs PC2) obtained by performing PCA on the MR spectra acquired on polar extracts (8 replicates per treatment condition) of PC3 and LNCaP prostate cancer cells. Control samples were compared to samples treated for 48 hours with either LY294002 ((A) for PC3 and (C) for LNCaP cells) or 17AAG ((B) for PC3 and (D) for LNCaP cells).

^1^H MR spectra were recorded on the polar fraction of the cell extracts (8 replicates per treatment per cell line) of PC3 and LNCaP prostate cancer cells. To investigate the effect of treatment on the metabolome of the two prostate cancer cell lines we performed multivariate statistical analysis on the complete MRS datasets either including both cell lines and all the treatment conditions or considering each individual cell line. The scores plots obtained from the analysis (**Fig. 2**) clearly highlight that, as expected, the two cell lines have extremely different phenotypes (more than 90% of the total variability is explained by the first principal component in **Fig. 2A**, segregating the two cell lines). Moreover, the scores plots obtained from the PCAs performed on the individual cell lines (**Fig. 2B–C**) demonstrate excellent clustering of samples within the same treatment group and clear separation between the 3 different treatment conditions (dimethyl sulfoxide (DMSO) as solvent control, LY294002 and 17AAG), for both PC3 and LNCaP cell lines. In light of the excellent separation indicated by the above analyses and with the objective of gaining a better understanding of the metabolic changes underlying the observed differences, we repeated the PCAs, performing comparisons of the spectra acquired on untreated (solvent control) samples versus those obtained from either LY294002 or 17AAG treated samples for each cell line. As expected, the scores plots obtained from these PCAs (**Fig. 3**) indicate the complete separation of solvent control and each of the treatments along the first principal component (PC1). The advantage of this analysis resides in the fact that the PC1 loadings plots capture the metabolic changes specifically induced by the considered drug treatment (compared to control). In fact, PC1 explains the large majority of the total variability (between 63% and 77% depending on the cell line/treatment considered), while PC2 explains less than 12% (between 9% and 12%) of the total variability.

**Figure 4:**
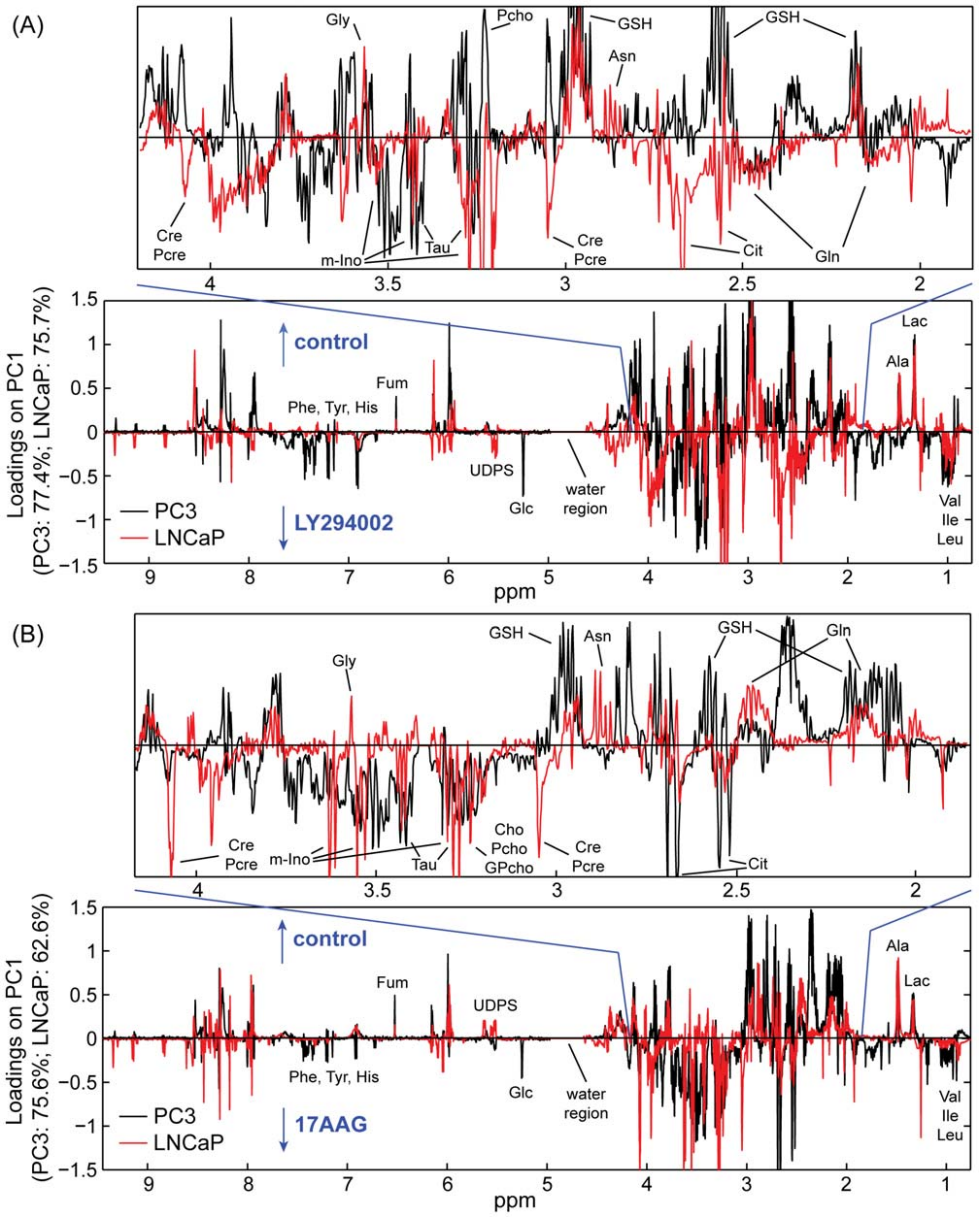
Multivariate statistical analysis of the MR spectra. Loadings plots (on PC1) obtained by performing the PCA comparisons on the MR spectra of control and one treatment per analysis (as shown in Fig. 3) acquired on polar extracts of PC3 (black line) and LNCaP (red line) prostate cancer cells following 48 hours of treatment with (A) LY294002 or (B) 17AAG. Enlarged sections of the loadings plots represent the region of 1.9–4.1 ppm. Ala: alanine; Asn: asparagine; Cho: choline; Cit: citrate; Cre: creatine; Fum: fumarate; Glc: glucose; Gln: glutamine; Gly: glycine; GPcho: glycerophosphocholine; GSH: glutathione; His: histidine; Ile: isoleucine; Lac: lactate; Leu: leucine; m-Ino: myo-inositol; Pcho: phosphocholine; Pcre: phosphocreatine; Phe: phenylalanine; Tau: taurine; Tyr: tyrosine; Val: valine, UDPS: UDP sugars.

The analysis of the loadings plots (**Fig. 4**) obtained from each of the PCAs revealed discriminatory metabolites for the different cell lines/treatments. Specifically, the loadings plots obtained from the comparison of solvent control and LY294002 treatments indicated the decrease of intracellular alanine, lactate, fumarate, glutathione and phosphocholine concentrations following treatment in both PC3 and LNCaP prostate cancer cells. The administration of LY294002 also induced the accumulation of branched amino acids (valine, leucine, isoleucine) and glutamine in both prostate cancer cells. Some disparate changes among the two prostate cancer cells were also observed, including the decrease of taurine, myo-inositol and uridine diphosphate (UDP)-glucose in PC3 cells and asparagine and glycine in LNCaP cells, and the accumulation of glucose, glycine, phenylalanine, tyrosine and histidine in PC3 cells and UDP-N-acetyl-glucosamine, UDP-N-acetyl-galactosamine, creatine, phosphocreatine, choline, glycerophosphocholine, taurine, myo-inositol and citrate in LNCaP cells.

**Figure 5:**
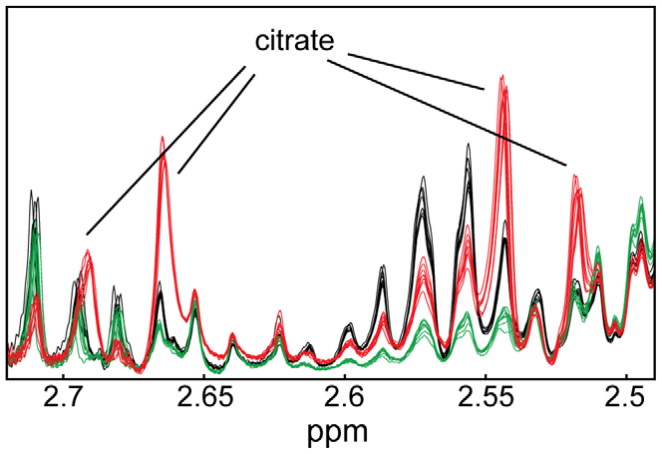
Accumulation of citrate following treatment with 17AAG in PC3 prostate cancer cells. Enlarged section (2.49 – 2.72 ppm) of the MR spectra acquired on polar extracts of PC3 cells following 48 hours of treatment with DMSO (solvent control, black), LY294002 (green) and 17AAG (red). Spectra were normalized according to the probabilistic quotient normalization method.

In the case of 17AAG treatment, a decrease in lactate, alanine, fumarate and glutamine, and the intracellular accumulation of valine, leucine, isoleucine, phosphocholine, myo-inositol, taurine and citrate were observed in both PC3 and LNCaP cells. It is worth noting that citrate represents the largest peak in the loadings plot, **Fig. 4B**, for PC3 cells treated with 17AAG. This metabolite was virtually undetected in untreated and LY294002-treated PC3 cells, but accumulated in substantial amounts following 17AAG treatment, as depicted in **Fig. 5**.

Other metabolic changes induced by treatment with 17AAG included the intracellular accumulation of glucose, glycine, phenylalanine, tyrosine and hystidine in PC3 and accumulation of creatine, phosphocreatine, choline and glycerophosphocholine in LNCaP cells. Concurrently glutathione and UDP-glucose decreased in PC3 and asparagine, glycine, UDP-N-acetyl-glucosamine and UDP-N-acetyl-galactosamine decreased in LNCaP cells. Interestingly, our results indicated that several metabolic changes in response to treatment with LY294002 and 17AAG were common to both prostate cancer cells.

### Targeted analysis of proton MR spectra of breast cancer cells

MCF-7 cells were treated with 25 µM LY294002 or 3 µM 17AAG. Similarly to the prostate samples, these doses were previously determined such that they induced inhibition of target proteins and inhibition of cell growth [24], [48].

**Figure 6:**
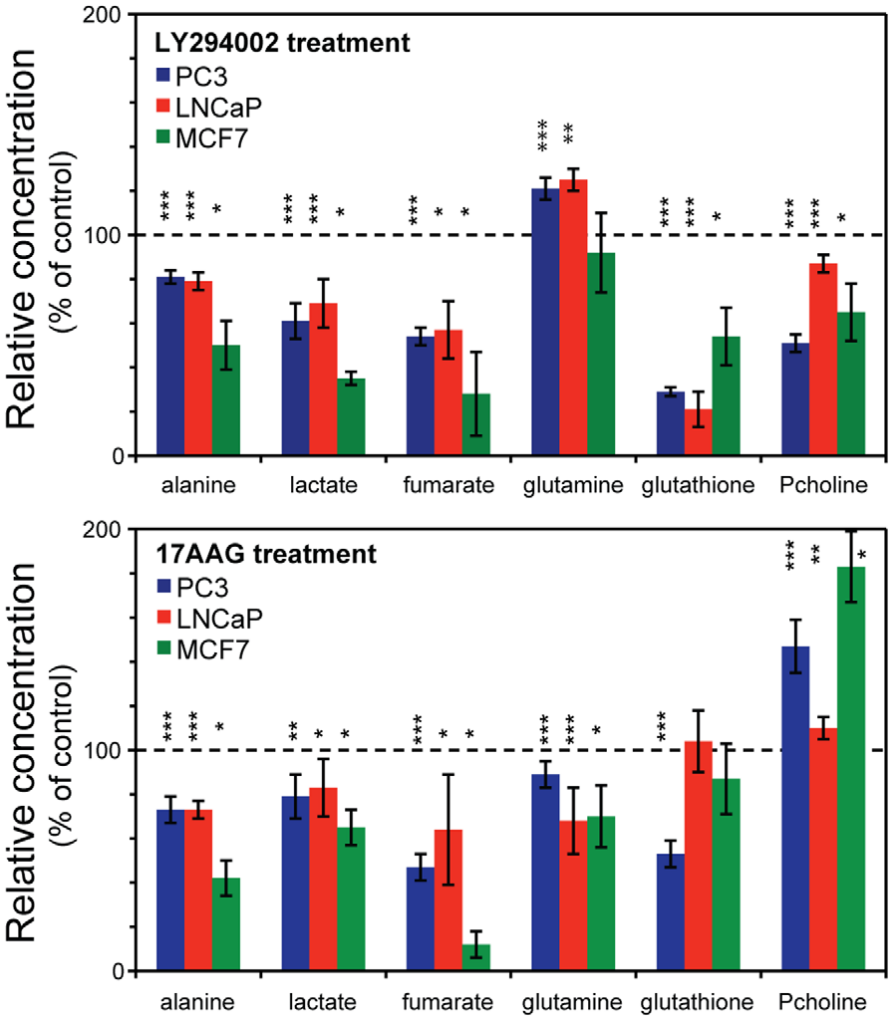
Common metabolic changes in prostate and breast cancer cells following drug treatment. Quantification of selected metabolites (shown as percent of control, mean ± standard deviation) from MR spectra acquired on prostate (PC3 and LNCaP, N = 8) and breast (MCF-7, N = 3) cancer cell lines following 48 hours of treatment with (A) LY294002 or (B) 17AAG. *: p<0.05; **: p<0.005; ***: p<0.0005. Pcholine: phosphocholine.

To confirm the generality of our findings in prostate cells we next conducted a *targeted* analysis of the metabolic consequences of LY294002 and 17AAG treatment in the MCF-7 cells. We focused on the common metabolic changes detected in the prostate samples when using the *untargeted* approach and therefore quantified the modulations in the intracellular levels of lactate, alanine, fumarate, phosphocholine, glutamine and glutathione. In complete agreement with the results reported above for the prostate cells, treatment with LY294002 induced the decrease of intracellular alanine, lactate, fumarate, glutathione and phosphocholine in MCF-7 breast cancer cells. Similarly, the decrease of lactate, alanine, fumarate and glutamine, and the accumulation of phosphocholine induced by the 17AAG treatment in PC3 and LNCaP cells was also confirmed in MCF-7 breast cancer cells. Quantification of the main metabolic changes is summarized in **Figure 6**.

## Discussion

In this study we investigated the global metabolic effects induced in PC3 and LNCaP prostate cancer cell lines through the pharmacological intervention with LY294002, a PI3K inhibitor, and 17AAG, an HSP90 chaperone-function inhibitor. We characterized the metabolic fingerprints of the prostate cancer cells with and without treatment using ^1^H MRS and then determined the metabolic changes associated with response to each treatment using a completely untargeted and unbiased multivariate statistical approach (PCA). Our aim was to identify the commonalities in the two cell lines of the metabolic signatures associated with response to treatment for each of the inhibitors. A common metabolic profile was observed in our studies that indicates that several metabolites are simultaneously modulated following treatment with each of the inhibitors. Moreover, we also confirmed the generality of the two treatment-specific metabolic signatures in a breast cancer cell line (MCF-7).

When considering the two inhibitors, both LY294002 and 17AAG have the ability to affect signaling via the PI3K pathway, as indicated by decreased p-4E-BP1 levels downstream of mammalian target of rapamycin (mTOR). The results of the metabolic profiling indicate that regardless of the inherently different metabolic fingerprint and genetic background of the two prostate and one breast cancer cell lines, changes in the metabolic signatures induced by the drug treatments demonstrate commonalities which are probably associated with the PI3K/Akt pathway and downstream modulation of HIF-1α. Both drug treatments induce the intracellular depletion of lactate and alanine in all 3 cell lines. Lactate dropped between 30% and 65% after LY294002 treatment and between 17% and 35% after 17AAG treatment, depending on the cell line. Alanine dropped between 20% and 50% with LY294002 and between 27% and 58% with 17AAG depending on the cell line. These results points to hindered glycolysis upon administration of the drug treatments and is consistent with the known activation of glucose uptake and glycolysis by PI3K/Akt signaling [49]. Intracellular fumarate also decreased (between 43% and 72% with LY294002 and between 36% and 88% with 17AAG treatment) in all prostate and breast cancer cells after the administration of either drug indicating that it might be a key mediator of this response. In fact, it has been previously reported that the inhibition of fumarate hydratase (FH) and the associated accumulation of intracellular fumarate coincide with HIF upregulation [50].

In contrast, other metabolites showed a “drug-specific” behavior. Phosphocholine dropped (13 to 50% depending on the cell line) following PI3K inhibition but increased (between 10 and 83%) following HSP90 inhibition, in line with previous studies. Phosphocholine was previously reported to decrease following treatment with LY294002 and wortmannin (another PI3K inhibitor; [22]) and increase following 17AAG treatment in human breast and colon cancer cell lines [24], [25].

The modulation of glutamine was also drug specific: it increased (approximately 25% in the 2 prostate cancer cell lines) or stayed constant following PI3K inhibition and dropped (20–30%) following HSP90 inhibition. To the best of our knowledge, these findings have not been previously reported. Whereas further studies are needed to fully understand the underlying mechanism of these changes, these observations are in line with the potentially fundamental role of glutaminolysis in cancer cell growth and the molecular/metabolic links reported between Myc and both glutaminase expression and glutamine-uptake regulation [51]–[53].

The observation that the intracellular concentration of citrate is increased following 17AAG treatment is highly significant in the context of prostate cancer as it might indicate a specific shift towards a more physiologic metabolism of prostate cancer cells. Citrate is known to be physiologically present in large amounts in the healthy prostate. However, citrate levels dramatically drop upon development of prostate cancer. Although 17AAG has not shown significant efficacy in a phase II clinical trial in prostate cancer patients, it is possible that the use of this drug combined with other agents would contribute to improving outcome by mediating a normalization of metabolism in prostate cancer cells.

In conclusion, this research highlights the potential of MRS-based untargeted metabolomics. Using this approach we identified a possible global metabolic signature associated with PI3K and HSP90 inhibition. This study not only confirmed previous work but also identified previously unknown putative links between signaling and metabolic pathways. Once individual metabolic changes are validated through detailed mechanistic studies, a combination of metabolic alterations could be envisaged which would provide a more specific signature of response than single metabolite biomarkers. Additional studies are under way in our lab in animal xenograft models of breast and prostate cancer to verify and validate *in vivo* the response signature to treatment with PI3K and HSP90 inhibitors using high resolution magic angle spinning (HR-MAS) MRS and MR spectroscopic imaging. The potential value of HR-MAS MR analysis of tumor biopsies to predict long-term survival and evaluate response to treatment has been previously reported [54]. In the long term, knowledge of the expected metabolic signature of response to targeted therapies could lead to the development of automated decision-support tools based on *in vivo* noninvasive patient MRS data similar to the approach developed in the context of the INTERPRET project (http://gabrmn.uab.es/INTERPRET) wherein the MRS signature is proposed as a tool to assist in the diagnosis and grading of brain tumors and other abnormal brain masses [55], [56]. Ultimately, this could lead to specific non-invasive methods for monitoring response in the *in vivo* clinical setting.

## Materials and Methods

### Cell culture and treatments

PC3, LNCaP (prostate) and MCF-7 (breast) cancer cell lines were obtained from American Type Culture Collection via University of California San Francisco (UCSF) Cell Culture Facility (San Francisco, CA, USA) and were maintained in exponential proliferation in Dulbecco's Modified Eagle Medium (DMEM) supplemented with 10% heat-inactivated fetal bovine serum, 2 mM L-glutamine, 100 units ml^−1^ of penicillin and 100 µg ml^−1^ of streptomycin. The cells were cultured in a humidified chamber at 37°C and with 5% CO_2_.

For all the experiments cells were incubated with drug for 48 hours as follows. PC3 cells with 25 µM LY294002 (PI3K inhibitor) and 1 µM 17AAG (HSP90 inhibitor), LNCaP cells with 10 µM LY294002 and 0.25 µM 17AAG and MCF-7 cells with 25 µM LY294002 and 3 µM 17AAG. The treatment doses were determined such that they decreased cell viability to approximately 50% of solvent control after 48 hours of treatment by using the cell proliferation assay detailed below. All treatments were performed with matching DMSO solvent control (1∶1000 final concentration in culture medium) and were replenished after 24 hours.

### Cell viability assay

The effect of different drug treatment doses on cell viability was determined using the WST-1 reagent assay (Roche). Cells were seeded in 96-well plates and treated for 4 to 48 hours with 4 different treatment doses for each drug. After treatment, WST-1 reagent was incubated in wells for approximately 1 hour and cell viability was determined by spectrophotometric (Tecan) quantification of absorbance at 440 nm.

### Western Blotting

The effect of treatment with LY294002 and 17AAG on the levels of target proteins was analyzed by Western blotting. Whole cell lysates from treated and untreated cells were separated on 4% to 20% SDS-PAGE gels (Bio-Rad). Proteins were then transferred onto nitrocellulose membranes, blocked and incubated with primary and secondary (anti-IgG horseradish peroxidase-linked, Cell Signaling) antibodies. Primary antibodies against p-4E-BP1, c-Raf and β-actin (as loading control) (Cell Signaling) were used. Immunocomplexes were visualized using enhanced chemiluminescence (ECL) Western Blotting Substrate (Pierce).

### MR sample preparation

In all cases, cells were extracted using a dual phased extraction, as described previously [29]. The extraction of MCF-7 breast cancer cells (3 replicates) was previously described in detail [24], [48]. In the case of PC3 or LNCaP prostate cancer cells, following 48 hour treatments, approximately 5×10^6^ cells were washed twice with phosphate buffered saline (PBS) in the tissue culture flask and then fixed using ice-cold methanol. Cells were then scraped off and transferred with the methanol to a glass centrifuge tube. Chloroform and water were then added to the methanol in equal volumes (final solution 1∶1∶1 methanol:chloroform:water). The solution was vortexed and centrifuged to separate the aqueous and lipid phases. The two phases were then collected separately and dried. The dried polar extracts (8 replicates per treatment per cell line) were then redissolved in 600 µl of 100 mM phosphate buffer (pH 7.0) prepared in 90% H_2_O - 10% D_2_O and containing 0.5 mM sodium 3-(trimethylsilyl)propionate-2,2,3,3-d4 (TMSP, Cambridge Isotope Laboratories) as internal reference.

### MR data acquisition and processing

One dimensional (1D)^ 1^H MR spectra acquisition was performed on the aqueous fraction of the PC3 and LNCaP cell extracts using a 600 MHz spectrometer equipped with a cryogenically cooled probe. 90° pulse and 4 s relaxation delay were used and the water resonance was suppressed using excitation sculpting [57]. The acquisition of ^1^H MR data from MCF-7 cells extracts was previously described [24], [48].

All the MRS datasets were processed using NMRLab [58] in the MATLAB programming environment (The MathWorks, Inc.). Following standard processing steps, spectra were aligned, selected signals arising from residual solvents (water, methanol and chloroform) and from TMSP were excluded. Spectra were normalized according to the probabilistic quotient method [59]. Spectra acquired on PC3 and LNCaP cell samples were then binned at approximately 0.0017 ppm and the generalized-log transformation was applied prior to conducting the multivariate statistical analysis [60]. Principal component analysis (PCA) of the complete PC3 and LNCaP extract MRS datasets was carried out using MATLAB. For all datasets, MRS resonances of metabolites were assigned by comparison with spectra of standard compounds (www.bml-nmr.org) and the peak integrals of selected metabolites were calculated using ACD/Spec Manager version 9.15 software (Advanced Chemistry Development) for relative quantification. Data are reported as mean values ± standard deviation. It should be noted that the MRS datasets were acquired under slightly different conditions for the breast and the prostate cancer samples. However, for each cell line the spectra of treated samples were always acquired under the exact same conditions as the matching control samples. The relative quantifications (as percent change when treatment is compared to control) are thereby unaffected by the differing acquisition conditions. For the prostate cancer samples (N = 8 per treatment condition and per cell line) statistical significance was determined using a Mann-Whitney U test with p<0.05 considered significant. For the breast cancer samples (N = 3) which were used to confirm the trends observed in the prostate samples, statistical significance was assessed using a one-sided Mann-Whitney U test with p<0.05 considered significant.
